# Construction of 1D Heterostructure NiCo@C/ZnO Nanorod with Enhanced Microwave Absorption

**DOI:** 10.1007/s40820-021-00704-5

**Published:** 2021-08-16

**Authors:** Jianwei Wang, Zirui Jia, Xuehua Liu, Jinlei Dou, Binghui Xu, Bingbing Wang, Guanglei Wu

**Affiliations:** 1 State Key Laboratory of Bio-Fibers and Eco-Textiles, Institute of Materials for Energy and Environment, College of Materials Science and Engineering, Qingdao University, Qingdao, 266071 People’s Republic of China; 2 College of Chemistry and Chemical Engineering, Qingdao University, Qingdao, 266071 Shandong People’s Republic of China; 3 Weihai Innovation Institute, Qingdao University, Weihai, 264200 Shandong People’s Republic of China

**Keywords:** Layered double hydroxides, NiCo@C/ZnO, Interface polarization, Microwave absorption

## Abstract

Layered double hydroxides (LDHs) have a special structure and atom composition, which are expected to be an excellent electromagnetic wave (EMW) absorber. However, it is still a problem that obtaining excellent EMW-absorbing materials from LDHs. Herein, we designed heterostructure NiCo-LDHs@ZnO nanorod and then subsequent heat treating to derive NiCo@C/ZnO composites. Finally, with the synergy of excellent dielectric loss and magnetic loss, an outstanding absorption performance could be achieved with the reflection loss of − 60.97 dB at the matching thickness of 2.3 mm, and the widest absorption bandwidth of 6.08 GHz was realized at 2.0 mm. Moreover, this research work provides a reference for the development and utilization of LDHs materials in the field of microwave absorption materials and can also provide ideas for the design of layered structural absorbers.

**Supplementary Information:**

The online version contains supplementary material available at 10.1007/s40820-021-00704-5.

## Highlights

- NiCo-LDHs successfully grow in situ on rod-like ZnO with unique structures.
- The interface of NiCo@C and rod-like ZnO result in interfacial polarization.
- The RLmin of NiCo@C/ZnO reaches − 60.97 dB with the EABmax of 6.08 GHz.
- The excellent performance comes from the effect of dielectric loss and magnetic loss.

**Supplementary Information:**

The online version contains supplementary material available at 10.1007/s40820-021-00704-5.

## Graphical abstract

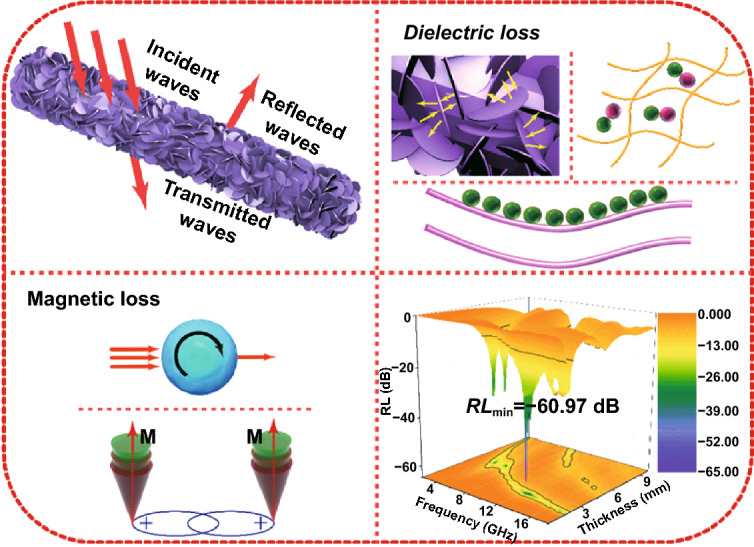

## Introduction

Nowadays, electromagnetic waves (EMW) are currently used in a wide range of medical and military equipment as a medium for the wireless control and transmission of information in electronic devices [1–3]. However, the failure of electronic facilities, human organ damage and other negative effects caused by electromagnetic pollution deserve our attention [4, 5]. In order to make better prevention of EMW, EMW-absorbing materials that can attenuate EMW energy and convert incident EMW into heat or otherwise forms of energy have been designed and developed [6, 7]. The idealized absorbing material should meet the requirement of wide bandwidth, light weight and thin thickness [8–10]. However, the EMW absorption capacity of a single material is relatively weak. Therefore, the development of composites that gratify the demands will become the focus of future studies.

Many suitable absorbers have been developed, such as carbon materials [11], alloys [12], transition metal oxides/sulfides/selenides [13–15], and MXene [16]. With the development of technology, the requirements for the EMW absorption properties and the condition of using EMW-absorbing materials are become more and more stringent. So, it is necessary to develop better materials to meet the requirements of practical application. At this point, metal alloys came into the research range due to excellent permittivity and permeability, with NiCo alloys being the most notable [17, 18]. NiCo alloys possess the characteristics of high-temperature calcination resistance, oxidation resistance, corrosion resistance, and easy extraction [19, 20], and are widely used in high-tech fields such as aerospace.

Metal organic frameworks (MOFs) are a new type of material formed by the self-assembly of metal cations with organic ligands, which have the advantages of large surface area, low density, and high porosity [21–24]. However, individual MOFs have not yet been extensively applied in EMW-absorbing materials as a result of poor dielectric properties [25]. Therefore, many attempts have been made to tune the electromagnetic properties of MOF materials and derivatives to obtain satisfactory EMW absorption performance. The most popular approach is introducing other metal cations to modulate the morphology and elemental composition of MOFs and derivatives [26, 27]. Che et al*.* [28] prepared Ni/C/ZnO microspheres with yolk-shell structure based on bimetallic NiZn-MOFs. Zhou et al*.* [29] prepared CoZn-MOFs as templates to prepare rod-shaped Co/ZnO/C composites, all of which can obtain excellent EMW-absorbing properties. However, there are still some difficulties, such as complicated preparation process and poor attenuation performance, etc. Therefore, it is still necessary to find other ideas to obtain ideal EMW-absorbing materials [30–32].

Layered double hydroxides (LDHs) are compounds consisting of positively charged lamellae and interlayer anions interacting with each other [33–35]. Generally, the most common methods for generating LDHs include hydrothermal method, ion exchange method, co-precipitation method, and so on [36, 37]. Metallic alloys are acquired via high-temperature calcination of LDHs materials in an inert gas environment, which is one of the methods to attain polymetallic alloys. The method is an important way to access excellent electromagnetic absorbing materials and make the electromagnetic parameters of the alloys controllable [38, 39]. Zinc oxide (ZnO) is a semiconductor material with a wide band gap and excellent dielectric properties, which, together with its ease of making unique structures, makes ZnO promising for development as dielectric material [40, 41].

In this paper, we presented a novel design that exploits the synergistic effect between components, which to develop electromagnetic absorbing materials that can meet practical needs. Hence, NiCo@C/ZnO composites decorated with NiCo alloy particles were synthesized through hydrothermal method and annealing process, which demonstrated excellent EMW absorption performance. The minimum reflection loss (*RL*_min_*)* value was − 60.97 dB with thickness of 2.3 mm, and the maximum effective absorption bandwidth (EAB_max_*)* value was 6.08 GHz with thickness of 2.0 mm. In addition, the electromagnetic absorption mechanism of NiCo@C/ZnO composites was discussed in detail. This work not only provides ideas for the development and utilization of MOFs materials, but also serves references for the design of new electromagnetic absorbing materials.

## Experimental Section

### Chemical Reagents

All chemicals were of analytical grade (AR) and used directly without any further purification. Cobalt (II) nitrate hexahydrate (Co(NO_3_)_2_·6H_2_O, AR, 99%), nickel (II) nitrate hexahydrate (Ni(NO_3_)_2_·6H_2_O AR, 99%), ammonia solution (NH_3_·H_2_O, 28%), Zinc acetate (Zn(CH_3_COO)_2_, AR, 99–101%), 2-methlimidazole (C_4_H_6_N_2_, 98%, 2-MIM), hexamethylene tetramine (C_6_H_12_N_4_), absolute ethanol (C_2_H_6_OH), and methyl alcohol (CH_3_OH, 99.5%). They are purchased from Chemical Reagents Company Limited of China Pharmaceutical Group. Deionized water was used throughout the experiment.

### Sample Preparation

#### Preparation of Rod-Like ZnO

**Scheme 1:**
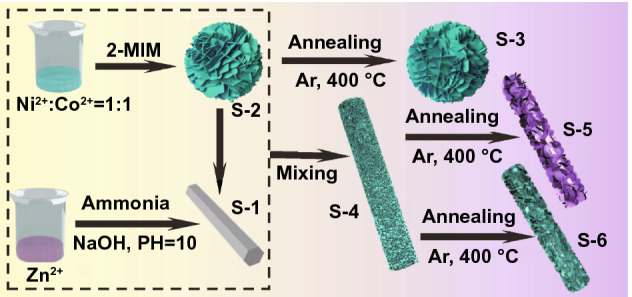
Illustration of the synthetic process of composites

As shown in Scheme 1, zinc acetate and hexamethylenetetramine were added to 50 mL of deionized water at the ratio of 1:1 and stirred with a glass rod until the particles were completely dissolved. The pH of the mixed solution was adjusted to pH = 10 with ammonia and stirred continuously at room temperature for about 3 h. The solution was transferred to 100 mL of PTFE liner and kept at 90 °C for 12 h. Finally, after cooling to room temperature, the sample was dried at 60 °C after washing with deionized water and ethanol. The rod-like ZnO materials were defined as S-1.

#### Preparation of NiCo@C Composites

0.5 g of Co(NO_3_)_2_·6H_2_O and 0.5 g of Ni(NO_3_)_3_·6H_2_O were added to 40 mL of methanol solution which was recorded as solution A. 8 mmol of 2-methylimidazole (2-MIM) was dissolved in 20 mL of methanol solution and stirred until the pellet was completely dissolved, which was recorded as solution B. Then, solution B was quickly poured into solution A, and stirred continuously at room temperature for 2 h. Then, the solution was transferred to a 100 mL PTFE liner and maintained at 160 °C for 12 h. After cooling to room temperature, the resulting material is washed several times with deionized water and ethanol and then dried at 60 °C. The samples were denoted as S-2. The material was calcined at 400 °C for 3 h under argon atmosphere at a heating rate of 5 °C min^−1^. The obtained composites were designated as S-3.

#### Preparation of Rod-Like Structured NiCo@C/ZnO Composites

Typically, 1.0 g of rod-like ZnO material is added to 40 mL methanol and stirred to obtain a homogeneous solution. 0.5 g of Co(NO_3_)_2_·6H_2_O and 0.5 g of Ni(NO_3_)_3_·6H_2_O were joined in the mixed solution. Then 8 mmol of 2-Methylimidazole (2-MIM) was dissolved in 20 mL of methanol solution with stirring until the pellet was fully dissolved. The 2-methylimidazole solution was quickly poured into a methanolic solution containing rod-like ZnO materials and stirred continuously for 2 h at room temperature. Then, the solution was transferred to a 100 mL PTFE liner and maintained at 160 °C for 12 h. After cooling to room temperature, the resulting material is washed several times with deionized water and ethanol and then dried at 60 °C. The samples were denoted as S-4. Finally, the composites were calcinated at 400 °C under Ar atmosphere and Air atmosphere with a heating rate of 5 °C min^−1^ for 3 h. The as-obtained composites were named as S-5 and S-6, respectively.

### Characterization

The crystalline structures of samples were obtained on powder X-ray diffraction (XRD, Rigaku Ultima IV with Cu-Ka radiation (*λ* = 0.15418)). The morphology of samples was characterized by a field emission scanning electron microscope (SEM, JFOL JSM-7800F) equipped with an energy-dispersive spectrum. The crystal structure and microstructure of samples were recorded by a transmission electron microscope (TEM, JEOL JEM-2100). Raman shift of samples was collected through using a Renishaw in Via Plus Micro-Raman spectroscopy system with a 50-mW laser at 532 mm. The bond states of surface elements for samples were tested by X-ray photoelectron spectroscopy (XPS) on Thermo Fisher ESCALAB 250Xi spectrometer with an Al Ka X-ray source (1486.6 eV). The thermogravimetric analysis (TGA) of samples was recorded by an SDT Q600 analyzer from room temperature to 900 °C with a heating rate of 10 °C min^−1^ under Ar atmosphere.

### Electromagnetic Parameters

Based on the mass ratio of 1:2, the composites and paraffin are mixed evenly, and pressed into a ring sample to be tested, in which the inner diameter, outer diameter, and thickness are 3.04, 7.00, and 2.00 mm, respectively. The EM parameters were obtained by a vector network analyzer (N5234A, Agilent, USA) using a coaxial method in the frequency range of 2.0–18.0 GHz. Using transmission line theory is to work out the reflection loss (*RL*) values of absorbers at different thicknesses in their corresponding frequency [42, 43].

1 $$Z_{{{\text{in}}}} = Z_{0} \sqrt {\mu_{r} /\varepsilon_{r} } \tanh \left| {j\left( {\frac{2\pi fd}{c}} \right)\sqrt {\varepsilon_{r} \mu_{r} } } \right|$$

2 $$RL\left( {{\text{dB}}} \right) = 20\log \left| {\frac{{Z_{{{\text{in}}}} - Z_{0} }}{{Z_{{{\text{in}}}} + Z_{0} }}} \right|$$

where *c*, *f* and *d* refer to the light speed, the corresponding frequency and the matching thickness, *Z*_in_ is the normalized input impedance and the *Z*_*0*_ means the impedance of free space, $\varepsilon_{r}$ and $\mu_{r}$ are the complex permittivity and complex permeability, respectively. Normally, when *RL* < − 10 dB, it means that the absorbing material will absorb more than 90% of the incident EMW.

## Results and Discussion

### Composition and Structure

**Fig. 1:**
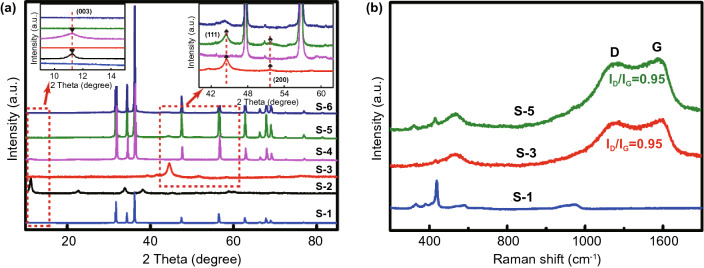
**a** XRD patterns of all samples, **b** Raman spectrum of S-1, S-4, and S-5

The crystal structures of all samples were analyzed by XRD. As displayed in Fig. 1, the curve of S-1 shows the XRD pattern of rod-like ZnO with characteristic peaks of 31.8°, 34.4°, 36.3°, 47.5°, 56.6°, and 62.9°, 66.4°, assigning to the (100), (002), (101), (102), (110), and (103), (200) of ZnO crystal planes. There are obvious characteristic peaks on the curve of S-2, where the characteristic peaks at 11.3°, 22.6°, 33.9°, 38.1° and 60.1° conform to the (003), (006), (100), (015), and (110) crystal planes of NiCo-LDHs, respectively [44]. The characteristic peaks corresponding to the (111), (200), and (220) crystals of Ni are 44.6°, 51.8° and 75.9°on the curves belonging to S-3 (PDF No. 4-0850). However, compared with the standard cards Co (PDF No. 15-0806) and Ni (PDF No. 4-0850), the position of the characteristic peak is somewhat shifted, which proves that the nickel–cobalt alloy is formed instead of a single metal. In Fig. S1, in addition to the characteristic peaks of NiCo alloys, characteristic peaks attributed to amorphous C can also be seen [11, 12], proving that NiCo@C can be obtained from NiCo-LDHs composites through high-temperature calcination in Ar. In the XRD spectrum of S-4, the characteristic peaks of NiCo-LDHs can be seen in addition to those of ZnO, which suggest the successful preparation of NiCo-LDHs@ZnO composites. NiCo@C/ZnO composites obtained via calcination of NiCo-LDHs@ZnO composites under Ar atmosphere are evidenced by the distinct peaks of ZnO and NiCo alloys in the XRD spectrum of S-5. The XRD spectrum of S-6 provides characteristic peaks of ZnO and Co_2_NiO_4_ from Co_2_NiO_4_@ZnO composites. No significant characteristic peaks of impurities were found in all the XRD patterns, confirming the high purity of the prepared samples. Figure 1b shows the Raman spectra of S-1, S-3, and S-5. The characteristic peak of ZnO (437 cm^−1^) appears on the curve of S-1. The characteristic peak of NiCo (534 cm^−1^) and obvious D/G peaks were noted in S-3, where the *I*_D_/*I*_G_ value was 0.95, which proves that graphitic carbon is produced during calcination at high temperatures. NiCo/C composites are favorable for the formation of conductive networks and the construction of heterogeneous interfaces. S-5 has both characteristic peaks of NiCo alloy, ZnO and obvious D/G peaks, in which the *I*_D_/*I*_G_ value does not change, and this conclusion is also consistent with the XRD results.

**Fig. 2:**
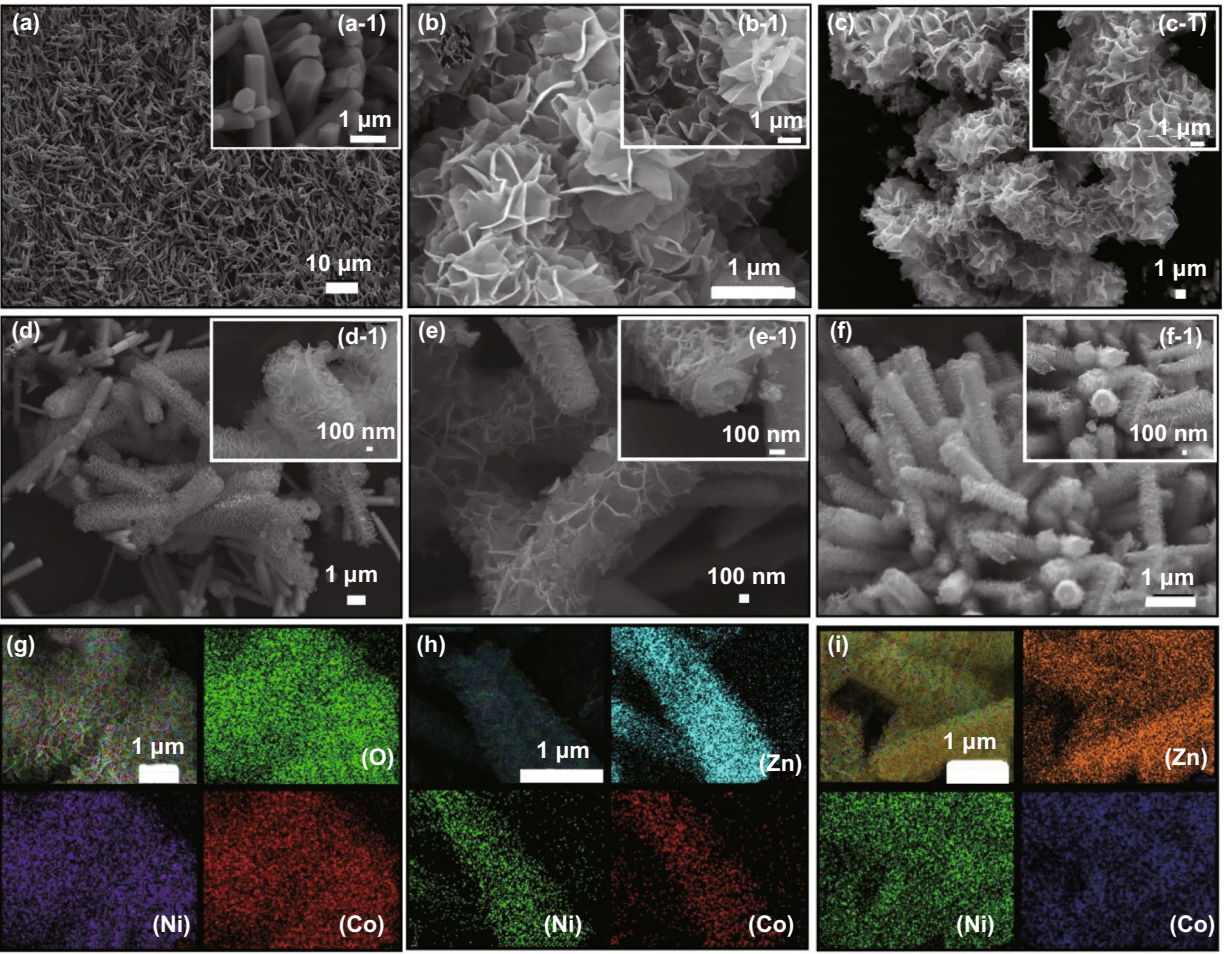
SEM images of (**a, a-1**) S-1, (**b, b-1**) S-2, (**c, c-1**) S-3, (**d, d-1**) S-4, (**e, e-1**) S-5, (**f, f-1**) S-6 and elemental mapping distribution of **g** S-2, **h** S-4, **i** S-5

The SEM images reveal the external morphology and elemental distribution of the sample. Figure 2a gives an SEM image of ZnO, which has a relatively homogeneous morphology and is not connected to each other. The cross section of the rod-shaped ZnO is ortho-hexagonal as seen in Fig. 2a-1. SEM image of NiCo-LDHs (S-2) (Fig. 1b) indicates the flower-like structures of NiCo-LDHs generated by stacking multiple lamellar structures, in which the size of each nanosheet is greater than 1 μm. It can be seen from the elemental distribution map of S-2 that the elements O, Ni and Co are uniformly dispersed in the NiCo-LDHs. The comparison of SEM images of S-2 (Fig. 2b) with S-3 (Fig. 2c) demonstrates no significant change in the overall morphology after high temperature annealing under Ar atmosphere, it underwent a transition from NiCo-LDH to NiCo@C, which led to the thinning of the nanosheets in S-3, the EDS spectrum (Fig. S2) indicate that this material is composed of three elements: nickel, cobalt and carbon, and their mass percentages are 38.42%, 33.96%, and 27.63%, respectively. In the SEM image of NiCo-LDHs@ZnO (S-4) (Fig. 2d), the surface of rod-like ZnO is covered with many layers of NiCo-LDHs and the layers are closely aligned. Zn elements are relatively concentrated, while Ni and Co elements are mainly scattered around the rod-like ZnO in the elemental distribution diagram. In Fig. 2e, the NiCo@C/ZnO composite (S-5) obtained after calcination at high temperature has a significantly lower number of layers coated on the rod-like ZnO. In the elemental distribution diagram (Figs. 2i and S3b), the distribution of Zn elements does not change obviously, while the distribution of Ni and Co elements becomes more dispersed and found that the contents of C, O, Ni, Co, and Zn were 15.03%, 14.43%, 14.76%, 11.92%, and 43.85% (Fig. S3b), respectively. The size of the layered structure of S-6 decreases significantly, but the number of layers remains almost unchanged (Fig. 2f).

**Fig. 3:**
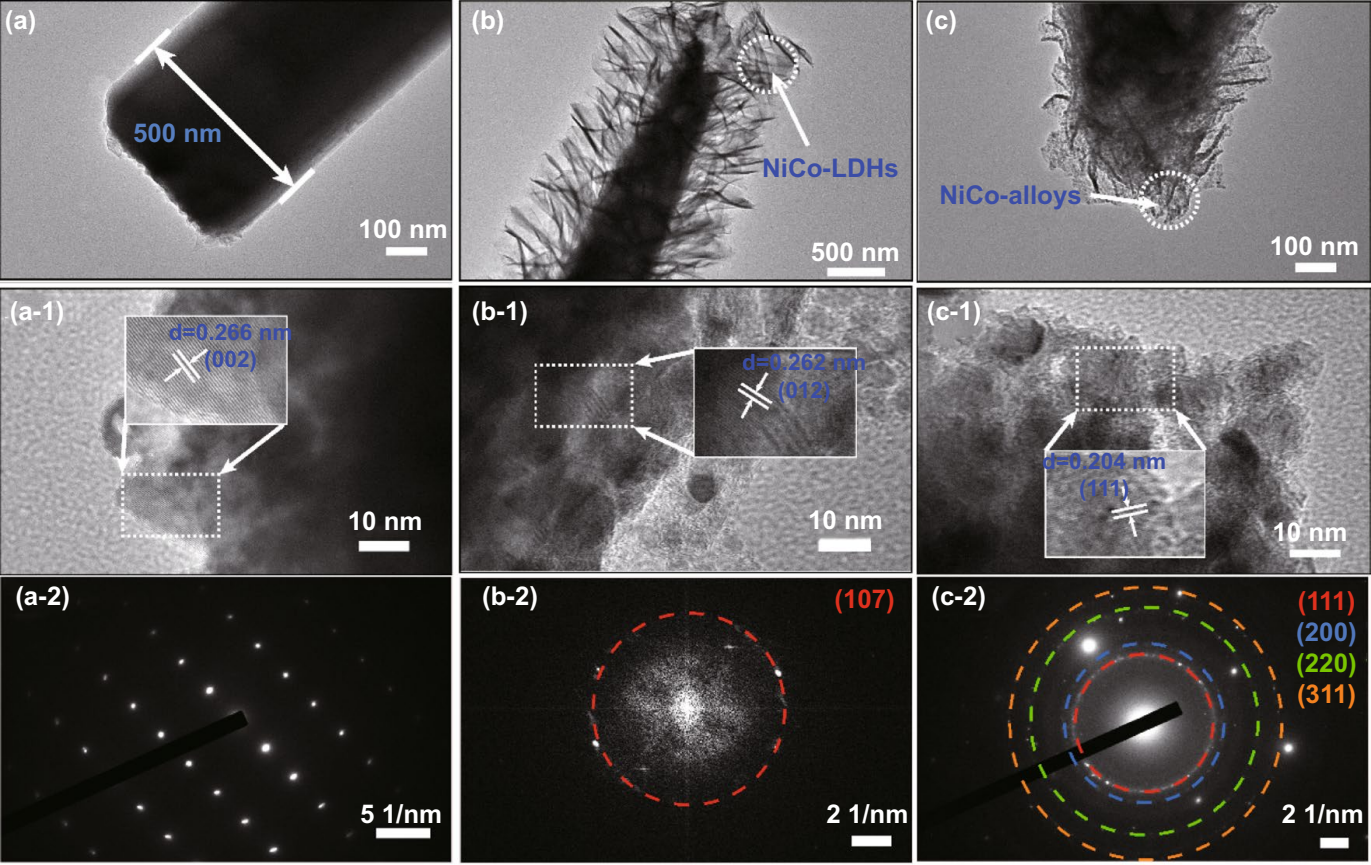
TEM images and SAED patterns of (**a, a-1, a-2**) S-1, (**b, b-1, b-2**) S-4, (**c, c-1, c-2**) S-5

The TEM images allow further to observe and analyze the internal structure and material composition of the prepared samples (Fig. 3). The rod-like ZnO exhibits a diameter of approximately 500 nm with no connections among themselves (Fig. 3a), which is consistent with observation in the SEM images (Fig. 2a). The lattice spacing (*d*) observed in the HR-TEM image of ZnO is 0.266 nm, matching the (002) crystal plane of ZnO (Fig. 3a-1). In Fig. 3a-2, the spots are uniformly dispersed and distributed in order, demonstrating the single-crystal hexagonal structure of ZnO. The layered structure overlying the rod structure can still be visualized in Fig. 3b, with the shaded part of the figure shows the NiCo-LDHs overlying the rod-like ZnO. The lattice with a spacing of 0.262 nm in Fig. 3b-1 corresponds to the (012) crystal plane of NiCo-LDHs [45]. The observed circle points to the (107) crystal plane of NiCo-LDHs in high-resolution TEM images of S-4 (Fig. 3b-2,). The lamellar structure changes in size and number after high-temperature annealing (Fig. 3c), and a number of grains (NiCo alloy) can also be observed. Besides, the lattice spacing of 0.204 nm (Fig. 3c-1) matches (111) plane of the NiCo alloy, which also confirms the formation of NiCo alloy [46, 47]. The diffraction rings in Fig. 3c-2 are attributed to the (111), (200), (220), and (311) crystal planes of the NiCo alloy, which are in accordance with the results of other characterizations.

**Fig. 4:**
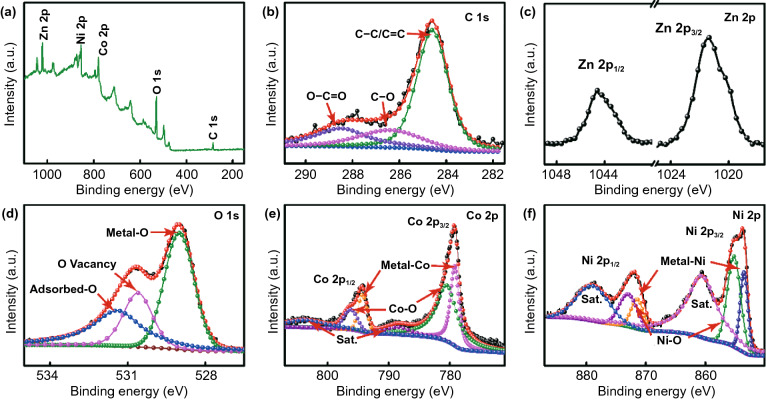
**a** Full scan XPS spectra of S-5, **b–f** high-resolution spectra C 1 s, Zn 2p, O 1 s, Co 2p, and Ni 2p of S-5

In order to further analyze the weight variation trend of the samples during calcination, TGA measurements were made in Ar atmosphere at a temperature increasing rate of 10 °C min^−1^ from room temperature to 900 °C. In Fig. S4, the mass of S-4 decreased slightly before 200 °C by about 0.99%, which is due to the gradual evaporation of the water contained in the sample with the increase in temperature. From 200 to 720 °C, the mass of the sample decreases linearly with the increase in temperature, and the final mass decreases to 13.05%, which was due to the gradual decomposition of NiCo-LDHs into NiCo@C composites with the increase in temperature. From the full-scan XPS spectrum (Fig. 4a), there are five characteristic peaks in S-5, which belong to C 1 s, Zn 2p, O 1 s, Co 2p, and Ni 2p, respectively. In the high-resolution spectra in C1 s (Fig. 4b), the characteristic peaks located at 284.6, 286.5, and 288.5 eV ascribed to C–C/C=C, C–O, and C–C=O, respectively [48]. From the high-resolution spectra of Zn 2p (Fig. 4c), the characteristic peak at 1021.4 eV corresponds to Zn 2p_3/2_ and the characteristic peak at 1044.6 eV corresponds to Zn 2p_1/2_. On the high-resolution spectrum of O 1 s of S-5 (Fig. 4d), three characteristic peaks of 531.5, 530.6, and 529.0 eV can be observed, corresponding to the water or O adsorbed on the sample surface, oxygen vacancies and metal-O (Ni–O/Co–O) respectively [49, 50]. Co 2p can be fitted to six distinct characteristic peaks (Fig. 4e), the characteristic peaks at 779.2 and 795.4 eV ascribed to metal-Co, and the characteristic peak at 780.6 and 796.3 eV can match the Co–O bond. The remaining characteristic peaks at 788.3 and 803.4 eV can be matched to the satellite peaks [51, 52]. In the Ni 2p high-resolution XPS spectrum of S-5 (Fig. 4f), the peaks at 878.9 and 860.8 eV are attributed to satellite peaks, and the peaks at 870.1 and 855.2 eV can be ascribed to the Ni–O bond. The O in the Co–O and Ni–O bond may originate from the surface oxidation of NiCo alloy when exposed to air. The characteristic peaks at 871.5 and 853.6 eV can be matched to the metal Ni in NiCo alloy [53, 54].

### Electromagnetic Performance and Parameter

**Fig. 5:**
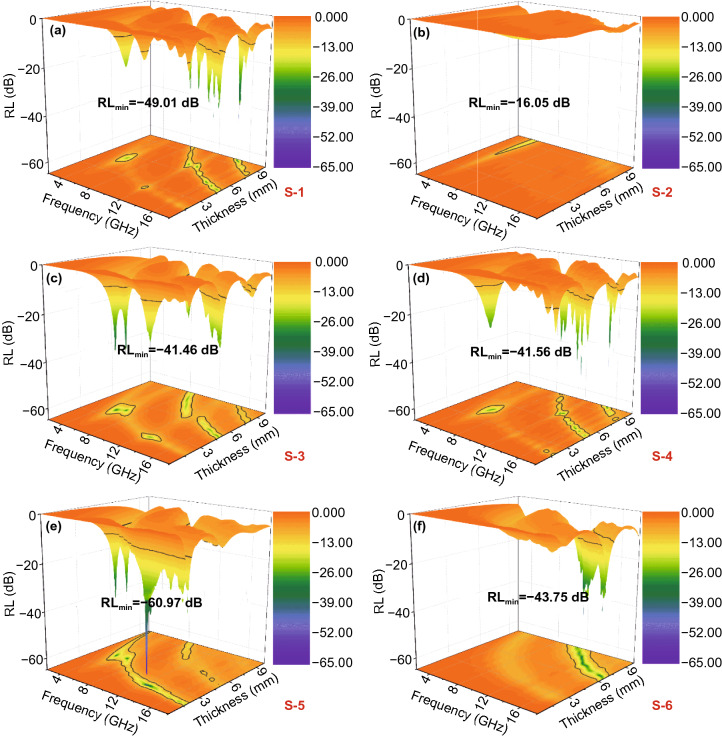
Reflection loss values in the frequency of 2–18 GHz for **a** S-1, **b** S-2, **c** S-3, **d** S-4, **e** S-5 and **f** S-6

**Fig. 6:**
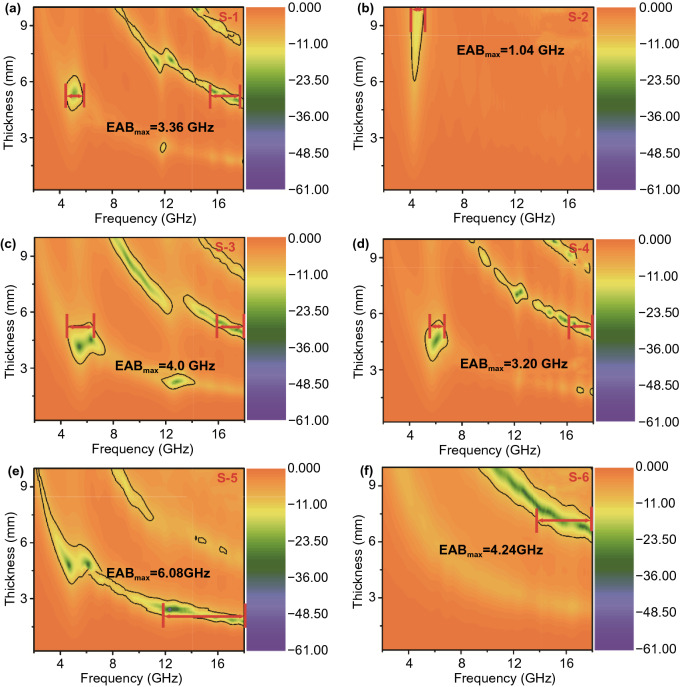
2D reflection loss diagram of **a** S-1, **b** S-2, **c** S-3, **d** S-4, **e** S-5, **f** S-6

According to Eqs. 1 and 2, we can calculate the relationship between the thickness of the corresponding EMW-absorbing material and the reflection loss (*RL*) in the range of 2–18 GHz frequency. The *RL*_min_ value is a key index to evaluate the performance of EMW-absorbing materials. Figures 5 and 6 show 3D and 2D reflection loss diagrams of frequency and thickness correspondence for all samples in the 2–18 GHz range. The *RL*_min_ value of S-1 is − 49.01 dB, corresponding to a thickness of 9.8 mm (Fig. 5a), and the EAB_max_ values reaches 3.36 GHz at 5.2 mm (Fig. 6a), which means the EMW absorption of S-1 performance is not ideal. Figures 5b and 6b present the EMW-absorbing performance of S-2. However, the *RL*_min_ value of − 16.05 dB and the EAB_max_ value of 1.04 GHz indicate that the EMW-absorbing performance of the NiCo-LDHs material also fail to meet the requirements. The corresponding *RL*_min_ value of S-3, at a thickness of 4.5 mm, is − 41.46 dB and the EAB_max_ values, at a thickness of 5.1 mm, is 4.0 GHz; the reason is that NiCo-LDH is transformed into NiCo@C composite by high-temperature annealing, which not only facilitates the construction of conductive network, but also the magnetic loss generated by NiCo alloy can improve the electromagnetic performance. In contrast, after plating the NiCo-LDHs on the surface of rod ZnO, the performance of S-4 has changed not much compared with S-1, the *RL*_min_ value of S-4 at 9.9 mm is − 46.51 dB, and the EAB_max_ value is 3.20 GHz, which corresponds to the thickness of 5.3 mm.

Figures 5e-f and 6e-f correspond to S-5 and S-6 obtained from calcining S-4 in different atmospheres, respectively. The results showed that the absorption properties of the products obtained by annealing in different inert atmospheres differed significantly. As compared to S-4, the EMW-absorbing performance of S-6 is slightly improved, and the *RL*_min_ value drops sharply at a thickness of 7.8 mm is − 43.75 dB, and the EAB_max_ value reaches 4.24 GHz at the thickness of 7.1 mm. However, the EMW absorption performance of S-5 is significantly increased. The *RL*_min_ value has reached − 60.97 dB, but the matching thickness is 2.3 mm. The EAB_max_ value has reached 6.08 GHz, and the matching thickness is only 2.0 mm. This is because the formation of NiCo@C composites makes significant changes in the structure of S-5, the increase in layer spacing makes the reflection and scattering of incident EMW more frequently, and the appearance of NiCo@C composites particles also promotes the polarization loss of incident EMW.

### Microwave Absorption and Mechanism

**Fig. 7:**
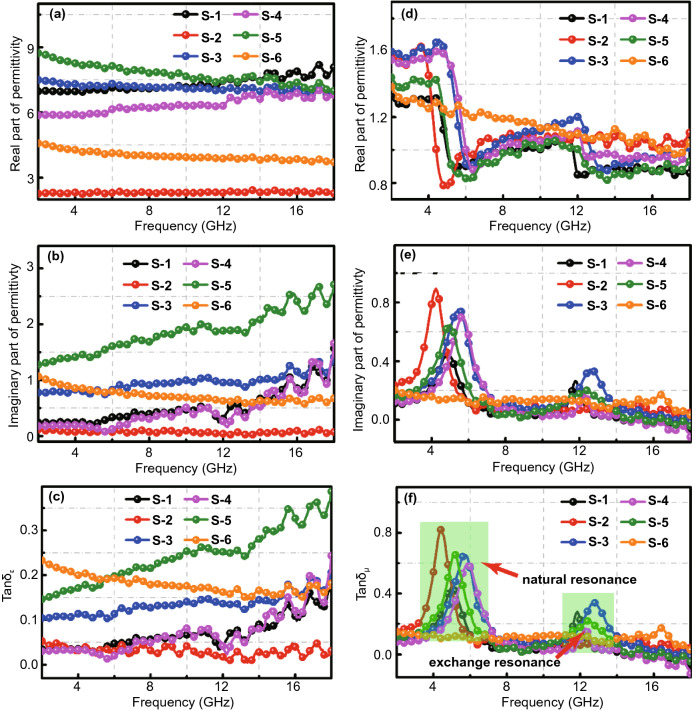
**a** Real part and **b** imaginary part of complex permittivity of all samples, **d** real part and **e** imaginary part of the complex permeability of all samples, **c** the dielectric loss tangent and **f** the magnetic loss tangent of all samples

The EM wave absorption characteristics of samples is usually analyzed according to the complex permittivity (*ε*_r_ = *ε*′ − *jε*″) and the complex permeability (*μ*_*r*_ = *μ*′ − *jμ*″) [55]. As shown in Fig. 7a, the real part of complex permittivity for S-5 is larger than that of other samples, and the *ε*′ value decreases gradually from 8.7 to 7.0 with the increase in frequency. It has typical dielectric response characteristics [56]. The *ε*′ values of S-1 and S-4 gradually increase with the increase in frequency, which is associated with the presence of ZnO. The *ε*′ values of S-2 and S-3 do not change significantly with frequency, but S-3 shows a significantly improvement than S-2. This is due to weak dielectric loss of NiCo-LDHs and NiCo alloy, which also proves that high-temperature calcination can significantly improve their dielectric properties. In addition, it can also be observed in Fig. 7b-c that the sample has multiple fluctuations at high-frequency bands, which implies that there is a significant polarization loss during the attenuation of the incident EMW, which could be due to the interfacial polarization caused by charge accumulation on heterogeneous junction surfaces and the dipole polarization occurring on defects or functional groups of materials. Both polarizations increase the dielectric loss capacity of the absorber [57, 58]. The curves of *μ*′ and *μ*″ versus frequency of all samples (Fig. 7d-e) were observed. A similar trend can be observed for all materials except for S-6, indicating poor magnetic properties. The *μ*′ values of all these materials occur in the range of 4–7 GHz. In general, the large fluctuations in the low-frequency band indicate the presence of natural resonance behavior, while fluctuations in the high-frequency band are attributed to exchange resonance, which contribute magnetic loss for the microwave-absorbing material [59, 60].

**Fig. 8:**
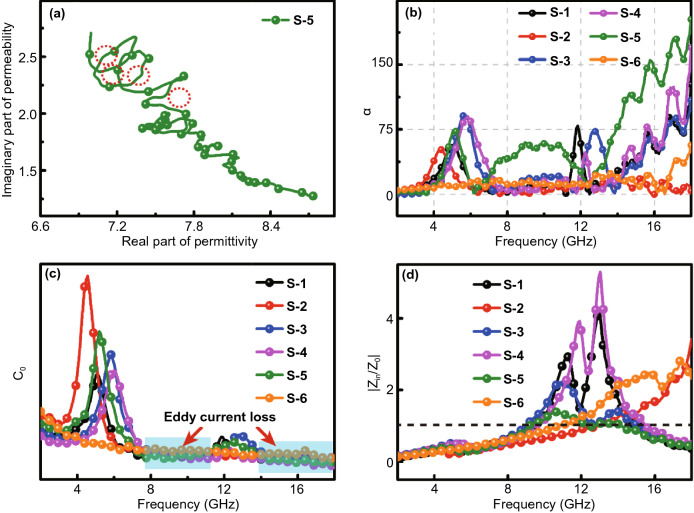
**a** Cole–Cole curves of S-5, **b** attenuation constant of all samples, **c** *C*_*0*_ of all samples, **d** impedance matching at 2.3 mm of all samples

Debye relaxation is an important way for absorbing materials to have dielectric loss. It can usually be deduced by Eqs. 3 and 4 to obtain Eq. 5, which can express relationship between *ε*′ and *ε*″ [61, 62].

3 $$\varepsilon^{\prime } = \varepsilon_{\infty } + \frac{{\varepsilon_{S} - \varepsilon_{\infty } }}{{1 + \left( {2\pi f} \right)^{2} \tau^{2} }}$$

4 $$\varepsilon^{\prime \prime} = \frac{{2\pi f\tau \left( {\varepsilon_{S} - \varepsilon_{\infty } } \right)}}{{1 + \left( {2\pi f} \right)^{2} \tau^{2} }}$$

5 $$\left( {\varepsilon^{\prime } - \frac{{\varepsilon_{S} + \varepsilon_{\infty } }}{2}} \right) + \left( {\varepsilon^{\prime \prime }} \right)^{2} = \left( {\frac{{\varepsilon_{S} - \varepsilon_{\infty } }}{2}} \right)^{2}$$

where *τ* stands for polarization relaxation time, *ε*_*s*_ for static permittivity, and *ε*_*∞*_ points to the high-frequency limited permittivity. As shown in Fig. 8a, more semicircles can be observed in the Cole–Cole diagram of S-5, which implies that more relaxation processes occur in S-5, related to the interface between ZnO@C and ZnO. The presence of a variable number of semicircles in other samples can also be seen in Fig. S5, which proves that the Debye relaxation process is prevalent in the prepared material [63]. According to the transmission line theory, the attenuation constant (*α*) can be calculated by Eq. 6 to reflect the attenuation ability of the absorbing material. Figure 8b is the correlation curve between attenuation coefficients and frequencies of all samples. With increasing frequency, all the curves show a significant change, but the attenuation system of S-5 is more prominent than that of other samples, which means that S-5 has a stronger attenuation ability to the incident EMW and shows better absorption performance, which conforms to our previous conclusion [64].

6 $$\alpha = \frac{\sqrt 2 \pi f}{c} \times \sqrt {\left( {\mu^{\prime\prime}\varepsilon^{\prime\prime} - \mu^{\prime}\varepsilon^{^{\prime}} } \right) + \sqrt {\left( {\mu^{\prime}\varepsilon^{\prime\prime} + \mu^{\prime\prime}\varepsilon^{\prime}} \right)^{2} + \left( {\mu^{\prime\prime}\varepsilon^{\prime\prime} - \mu^{\prime}\varepsilon^{\prime}} \right)^{2} } }$$

7 $$C_{0} = \mu^{\prime\prime}\left( {\mu^{\prime } } \right)^{ - 2} f^{ - 1} = 2\pi \mu_{0} d^{2} \delta$$

where *d* denotes the thickness of the absorber and *μ*_*0*_ means vacuum permeability. The relationship between *C*_*0*_ value and frequency can be used to analyze the mechanism of magnetic loss. When eddy current loss occurs inside the absorbing material, the *C*_*0*_ value will not change significantly with the change of frequency [65]. Figure 8c is the correlation curve between *C*_*0*_ and the frequency of all samples. As the frequency increases, the *C*_*0*_ value of S-6 does not change much, which indicates that the magnetic loss caused by eddy current loss plays a dominant role, while the other samples have two larger fluctuating peaks at 4–7 and 12–14 GHz, representing natural resonance and exchange resonance respectively. This is consistent with our previous analysis. In the range of 8–11 and 14–18 GHz, the variation of C_0_ value is quite small, which indicates that the eddy current loss plays a greater role in the attenuation of electromagnetic waves in this frequency range.

In addition to the attenuation coefficient, impedance matching ($\left| {Z_{{{\text{in}}}} /Z_{0} } \right|$) is a critical factor to determine the absorption performance of EMW--absorbing materials. According to Eq. 7, we can get the relationship between $\left| {Z_{{{\text{in}}}} /Z_{0} } \right|$ and frequency of different samples. Generally, the closer the $\left| {Z_{{{\text{in}}}} /Z_{0} } \right|$ value of the EMW-absorbing material is to 1, the EMW materials will absorb EM more easily [66], and the absorbing material will show better EMW absorption performance. From Fig. 8d, it can be found that the curve of S-5 is closer to 1, which matches its excellent EMW-absorbing performance. The impedance matching values of other materials vary greatly, which means that their impedance matching performance is poor, giving rise to poor EMW absorption performance [67].

**Scheme 2:**
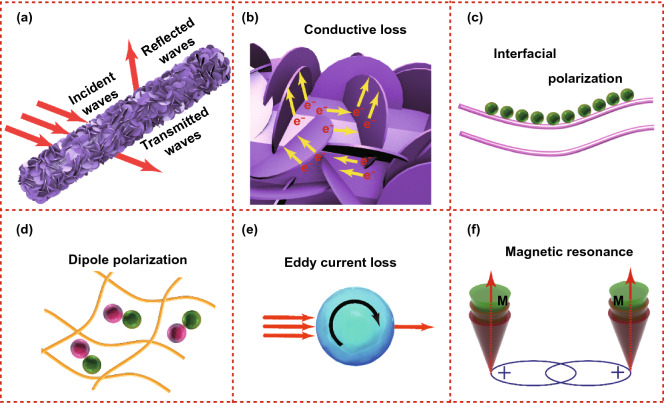
EM wave absorption mechanism of S-2

Scheme 2 shows a possible EMW absorption mechanism for S-5, which implies that the excellent absorption performance comes as a result of multiple mechanisms. Due to the large specific surface area of the composite material, it is easy to form a conductive network, which is conducive to induced current transmission under the action of the external magnetic field, causing the internal electrons to undergo directional migration and converting electromagnetic energy into thermal energy [68, 69]. On the one hand, the incident EMW that can enter the material is reflected and scattered, with large amount of EMW attenuating in the process [70]. Secondly, due to the different layered media between NiCo alloy and rod-like ZnO, electrons will accumulate on the contact interface, leading to interface polarization, which is an important reason for the excellent dielectric parameters of S-5 [71, 72]. Thirdly, the presence of NiCo@C composites and O vacancies cause dipole polarization in face of an external magnetic field, which promotes the loss of incident EMW. Furthermore, the eddy current loss and resonance loss caused by the presence of NiCo alloy are main sources of the magnetic loss [73]. In addition, as shown in Table 1, the composite absorber prepared in this study has better overall absorption performance than other composites.

**Table 1:** electromagnetic wave absorption performance of typical composites

Sample	*RL*_min_ (dB)	EAB (GHz)	*d* (mm)	References
Ni@C	− 59.5	4.7	2.5	[12]
Ni@C@ZnO	− 55.8	4.1	2.5	[28]
Co/ZnO/Ti_3_C_2_T_x_	− 44.22	5.28	2.4	[30]
Ni/NiO@C	− 51.1	5.12	2.7	[46]
C/MoS_2_	− 50.1	6.0	2.2	[58]
Fe/MnO@C	− 45.0	5.0	2.0	[60]
CNT-CoFe@C	− 40.0	5.62	2.0	[71]
NiCo@C/ZnO	− 60.97	6.08	2.0	This work

## Conclusion

In summary, we obtained NiCo-LDHs@ZnO composites through a reasonable combination of NiCo-LDHs with rod-like ZnO through simple experiments, and the final NiCo@C/ZnO composites were obtained after calcination and had excellent EMW absorption properties. The *RL*_min_ value reached − 60.97 dB at the matching thickness of 2.3 mm, and the EAB_max_ value is 6.08 GHz when the matching thickness is 2.0 mm. The interaction of dielectric and magnetic losses is the main reason for the excellent attenuation properties of EMW. This work provides an idea for further expanding the application of LDHs in absorbent materials and provides a method for preparing hetero-structured absorbent materials.

## Supplementary Information

Below is the link to the electronic supplementary material.

Supplementary file1 (PDF 674 KB)
