# Supplementary material for: Construction of 1D Heterostructure NiCo@C/ZnO Nanorod with Enhanced Microwave Absorption
Source: Nanomicro Lett. 2021 Aug 16;13:175. doi: 10.1007/s40820-021-00704-5 (PMC8368508; doi:10.1007/s40820-021-00704-5)
Supplement: Supplementary file 1 — Supplementary file1 (PDF 674 KB) [file 40820_2021_704_MOESM1_ESM.pdf]

Supporting Information for

Construction of 1D Heterostructure NiCo@C/ZnO Nanorod with Enhanced Microwave Absorption

Jianwei Wang[1, #], Zirui Jia[1, 2, 3, #], Xuehua Liu[1], Jinlei Dou[1], Binghui Xu[1], Bingbing Wang[1], Guanglei Wu[1, *]

[1]Institute of Materials for Energy and Environment, State Key Laboratory of Bio-fibers and Eco-textiles, College of Materials Science and Engineering, Qingdao University, Qingdao 266071, P. R. China

[2]College of Chemistry and Chemical Engineering, Qingdao University, Shandong, Qingdao 266071, P. R. China

[3]Weihai Innovation Institute, Qingdao University, Shandong, China 264200, P. R. China

[#]Jianwei Wang and Zirui Jia contributed equally to this work.

*Corresponding author. E-mail: wuguanglei@qdu.edu.cn or wuguanglei@mail.xjtu.edu.cn (G. Wu)

Supplementary Tables and Figures

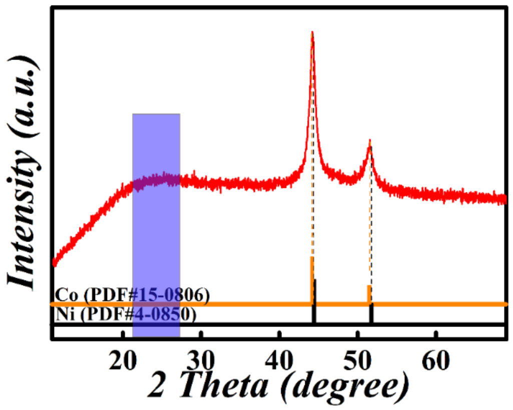

Fig. S1 XRD patterns of S-3

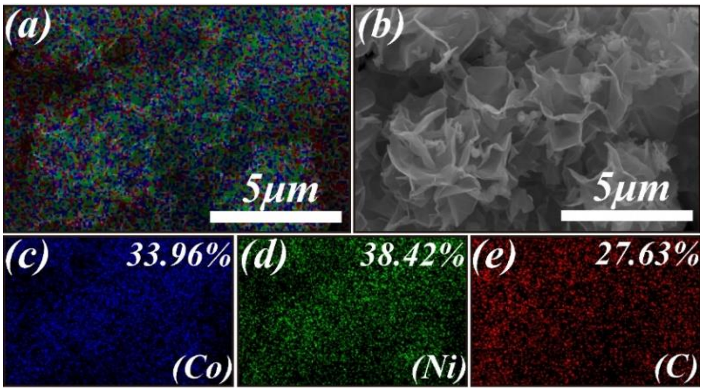

Fig. S2 elemental mapping distribution of S-3

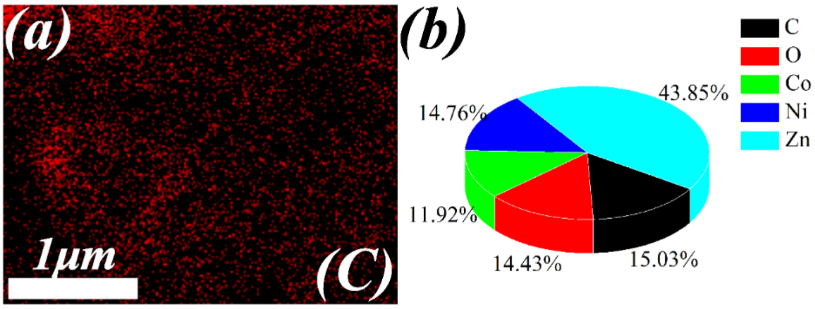

Fig. S3 (**a**) Carbon element distribution map. (**b**) Percentage of element in S-5

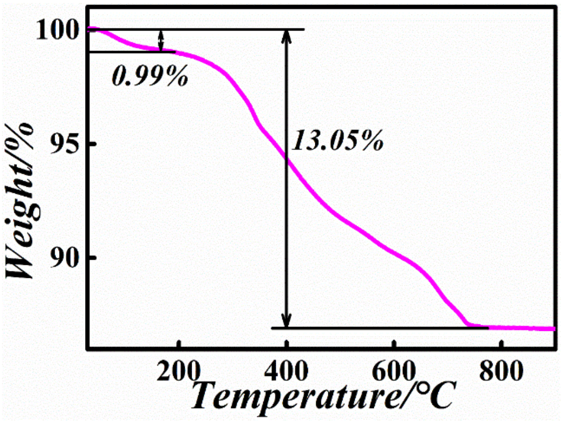

Fig. S4 TGA of S-4

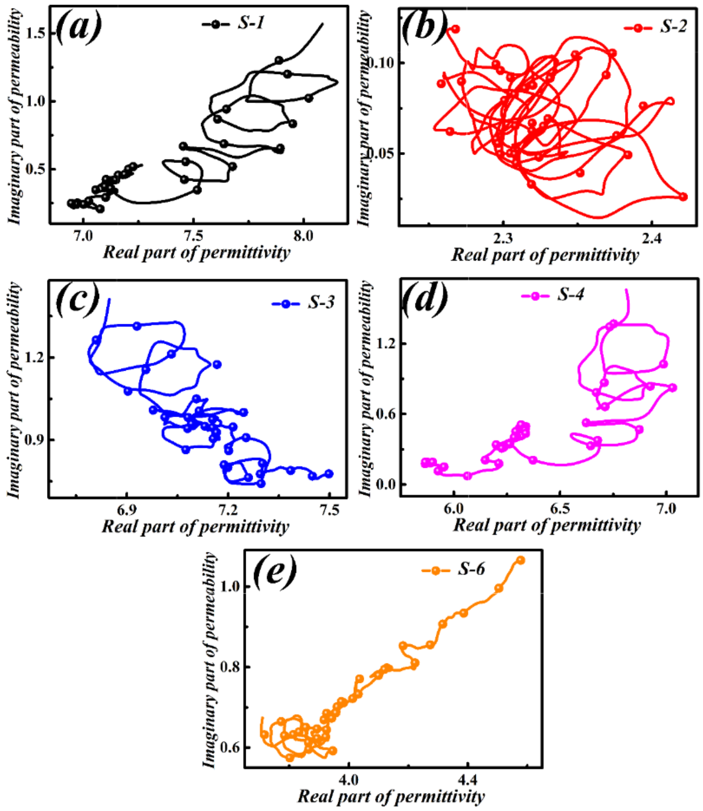

Fig. S5 Cole-Cole curves of (**a**) S-1, (**b**) S-2, (**c**) S-3, (**d**) S-4, (**e**) S-6
